# *Eubacterium rectale* contributes to colorectal cancer initiation via promoting colitis

**DOI:** 10.1186/s13099-020-00396-z

**Published:** 2021-01-12

**Authors:** Yijia Wang, Xuehua Wan, Xiaojing Wu, Chunze Zhang, Jun Liu, Shaobin Hou

**Affiliations:** 1 Laboratory of Oncologic Molecular Medicine, Tianjin Union Medical Center, Nankai University, No. 190 Jieyuan Rd., Hongqiao district, Tianjin, 300121 China; 2 TEDA Institute of Biological Sciences and Biotechnology, Nankai University, TEDA, Tianjin, 300071 China; 3 Advanced Studies in Genomics, Proteomics, and Bioinformatics, University of Hawaii At Manoa, 2538 McCarthy Mall, Snyder Hall, Honolulu, HI 96822 USA

**Keywords:** *Eubacterium rectale*, Colorectal cancer, ‘driver-passenger’ model, Colitis, Microbiota

## Abstract

**Background:**

Inflammatory bowel disease caused by microbial dysbiosis is an important factor contributing to colorectal cancer (CRC) initiation. The ‘driver-passenger’ model in human gut microbial dysbiosis suggests that ‘driver’ bacteria may colonize with low relative abundance on tumor site but persistently induce chronic change in normal intestinal epithelium and initiate CRC. They are gradually replaced by ‘passenger’ bacteria later on, due to their low adaptability to the on-tumor site niche.

**Results:**

To reveal site-specific bacterial taxon markers in CRC patients, we analyzed the gut mucosal microbiome of 75 paired samples of on-tumor and tumor-adjacent sites, 75 off-tumor sites, and 26 healthy controls. Linear discriminant analysis of relative abundance profiles revealed unique bacterial taxon distribution correlated with specific tumor sites, with *Eubacterium* having the distribution characteristic of potential driver bacteria. We further show that *Eubacterium rectale* endotoxin activates the transcription factor NF-κΒ, which regulates multiple aspects of innate and adaptive immune responses in normal colon epithelial cells. Unlike the ‘passenger’ bacterium *Fusobacterium nucleatum*, *E. rectale* promotes dextran sodium sulfate-induced colitis in Balb/c mice.

**Conclusions:**

Our findings reveal that *E. rectale* functions as a ‘driver’ bacterium and contributes to cancer initiation via promoting inflammation.

## Background

The adenoma-carcinoma sequence indicates that the accumulation of genetic and epigenetic alterations induces malignant epithelial cell proliferation, leading to colorectal cancer (CRC) [1]. Recent studies suggest that gut microbial dysbiosis results in colitis which contributes to initiation and development of CRC [2]. It is hardly to obtain samples from a same patient who went through the process from inflammatory bowel disease (IBD) to CRC, so distribution of gut bacteria in on-tumor sites and off-tumor sites of CRC patients is usually used to investigate bacteria associated with initiation of CRC by promoting inflammation. Deep sequencing-based 16S rRNA profiling can detect biodiversity of gut microbiome and reveal the relationship between gut microbiome and sporadic CRC. Microbial metabolites, such as butyrate and hydrogen sulfide [3, 4], as well as bacterial pathogen-associated molecular patterns (PAMP), such as lipopolysaccharide (LPS) [2], are known for triggering proinflammatory cascades that lead to adenoma-carcinoma sequence.

A variety of gut bacteria is more abundant on CRC tissue surface than on normal large intestinal surface. For example, *Fusobacterium nucleatum* in tumor samples shows high abundance but is almost absent from normal intestinal surface [5]. However, *F. nucleatum* may not promote CRC [6], because its high abundance in tumor samples may be caused by alterations of gut microenvironment [7]. *F. nucleatum* does not exhibit pro-tumorigenic or proinflammatory activities in preclinical models of colon carcinogenesis [6] and has high abundance in CRC samples, but not colorectal carcinoma [8]. Some bacteria, like *F. nucleatum,* cannot breach the intact healthy colon wall and colonize. However, they have a competitive advantage in tumor environment with rupture and bleeding of the colonic epithelium [7]. Thus, these bacteria are ‘passenger’ bacteria based on the ‘driver-passenger’ model proposed by Tjalsma et al. [9].

‘Driver’ bacteria initiate CRC by inducing IBD in epithelial cells. Meanwhile, they are gradually substituted by ‘passenger’ bacteria because gut microenvironment changes during tumor development. Many ‘driver’ bacteria are prone to be ignored if their abundances in CRC tissue are used as the only criterion. For example, *Bacteroides fragilis* is a potential ‘driver’ bacterium. It is a common colonic commensal which colonizes the majority of human guts. A subset of them is identified as enterotoxigenic *B. fragilis* (ETBF) which secrets a 20 kDa metalloprotease toxin (BFT). BFT cleaves E-cadherin, stimulates cell proliferation, and promotes IBD [10, 11]. Furthermore, CRC microbiomes analysis showed that *Bacteroides* are less abundant in CRC tissue than in adjacent non-malignant tissue [5]. Thus, as a potential ‘driver’ bacterium, *B. fragilis* secrets a toxin that may contribute to IBD that leads to CRC initiation, but it has relatively low growth competition on CRC epithelium. Bacterial taxon markers associated with off-tumor sites may serve as potential driver bacteria for CRC initiation [9]. Further identification and characterization of driver bacteria and their pathogenic activities will pave the way to CRC prevention therapies.

To identify CRC-associated gut microbiome profiles and potential driver bacteria that initiate CRC, we performed 16S ribosomal RNA gene sequencing on gut mucosal microbiome of paired samples of tumor and tumor-adjacent mucosae, off-tumor sites and healthy controls. The gut microbiome of tumor mucosae, but not that of tumor-adjacent mucosae and healthy control, shows lower alpha-diversity and distinct bacterial taxa compared with that at off-tumor sites. Linear discriminant analysis Effect Size (LEfSe) revealed specific bacterial taxa associated with tumor and tumor-adjacent mucosae, off-tumor sites and healthy controls, respectively. Moreover, the relative abundance of *Eubacteriaceae* is higher on off-tumor sites than tumor mucosae. Using *Eubacterium rectale* as an example, we showed the effect of *E. rectale* LPS on colon epithelium cells in vitro and that *E. rectale* promotes IBD in vivo.

## Materials and methods

### Reagents

All cell culture media, trypsin and antibiotics were purchased from Gibco (Grand Island, NY, USA), and FBS was purchased from HyClone (Logan, UT, USA). DAPI, lysosyme, proteinase K, DNase and RNase were purchased from Sigma-Aldrich (St Louis, MO, USA). Immobilon membranes were purchased from Merck Millipore (Bedford, MA, USA). ECL Plus substrate was purchased from CWBio (Beijing, China). Nuclear and cytoplasmic protein extraction kit was purchased from Beyotime (Shanghai, China). ZR Fungal/Bacterial DNA Kit was purchased from Zymo Research Corp. (Irvine, CA, USA). Qualitative fecal occult blood detection kit was purchased from Beijing Leagene Biotechnology Co., Ltd. (Beijing, China). Limulus Amebocyte Lysate (catalogs: T7572) was purchased from Solarbio (Beijing, China).

Rabbit anti-nuclear factor kappa B (anti-NF-κΒ) p65 antibody (catalogs: SAB4502610), rabbit anti-Histone H3 antibody (catalogs: SAB4500354), rabbit anti-IKKα antibody (catalogs: SAB4500257), rabbit anti-IκBα antibody (catalogs: SAB1305978), rabbit anti-β-actin antibody (catalogs: SAB2100037), goat anti-rabbit IgG-peroxidase (catalogs: A0545) and goat anti-rabbit IgG FITC (catalogs: AP132F) were purchased from Sigma-Aldrich (St Louis, MO, USA).

### Sample collection and preparation

Intestinal microflora samples were collected from 75 patients and 26 health people in Tianjin Union Medical Center. Cotton swab was used to dip on intestinal surface of colorectal cancer tissues collected from patients. Three distinct locations from the same tissue of a given cancer patient were collected, including tumor position (T), para-tumor epithelia position (P) and normal epithelia position (N). Intestinal microflora samples of healthy people were collected when people were diagnosed for colonoscopy and determined as healthy people (H). These samples were rinsed in 1 ml physiological saline. Then, 200 μl of these solutions were used for Bacterial DNA extraction. Bacterial DNA of these samples was extracted using ZR Fungal/Bacterial DNA Kit according to the manufacturer’s instructions.

### 16S RNA sequencing and bioinformatic analysis

The 16S ribosomal RNA amplicon libraries were constructed according to the Illumina manufactory manual. Briefly, the following primers were used to amplify the V3-V4 region of 16S rRNA gene: forward primer, 5′TCGTCGGCAGCGTCAGATGTGTATAAGAGACAGCCTACGGGNGGCWGCAG and reverse primer 5′GTCTCGTGGGCTCGGAGATGTGTATAAGAGACAGGACTACHVGGGTATCTAATCC.

The amplified DNA library was subsequently purified using AMPure XP beads (Beckman Coulter, USA) and quantified using Quant-iT PicoGreen dsDNA assay kit (Thermo Fisher, USA). The paired-end sequencing reads (2 × 300 bp) were generated on Illumina MiSeq platform, according to the Illumina standard protocol. Quality control and filtering of raw sequences were carried out using FastQC (https://www.bioinformatics.babraham.ac.uk/projects/fastqc/). The filtered paired-end reads were assembled using PandaSeq [33]. The assembled sequences were loaded on QIIME pipeline (qiime.org) [34] for de novo OTU picking, taxonomic assignment, and diversity analyses. Usearch was used within QIIME to detect and remove de novo chimera. Rarefaction was performed using alpha_rarefaction.py in QIIME pipeline. De novo OTUs were picked based on sequence similarity (97%) within the assembled sequences. Next, taxonomy was assigned to OTU representative sequences that were picked for each OTU. To identify differential abundance of bacterial phylo associated with specific sites of CRC patients and healthy people, we applied LEfSe [35].

### Bacterial strains and culture conditions

*E. rectale* ATCC 33656 and *F. nucleatum subsp. Nucleatum* ATCC 25586 were both purchased from Biobw (Beijing, China). *E. rectale* was grown anaerobically in PYG medium (ATCC medium 1527) composing of 0.5% peptone, 1% yeast extract, 0.5% tryptone, 0.01% resazurin (Sigma), 0.0008% MgSO_4_, 0.0008% anhydrous CaCl_2_, 0.004% K_2_HPO_4_, 0.004% KH_2_PO_4_, 0.002% NaCl, 0.04% NaHCO_3_, 0.0001% (v/v) Vitamin K1 (Sigma), 0.05% L-cysteine-HCL (Sigma). The pH was adjusted to 7.0. *F. nucleatum* ATCC 25586 was maintained anaerobically in Columbia broth (BD Biosciences). PYG medium and Columbia broth were both boiled to remove oxygen and placed in anaerobic bottles. Argon gas was added to the bottles. Then the bottles were sealed by a cap with a rubber septum before being autoclaved for 20 min at 121 °C.

### Mouse models of bacterial colonization

Colitis was experimentally induced by administration of 2.5% Dextran sodium sulfate (DSS) in the drinking water of 7-week-old Balb/c mice for 7 days. Afterwards, the mice were allowed to drink water without DSS until the end of experiment. After 2 days of DSS drinking, mice were inoculated with *E. rectale* or *F. nucleatum* by coloclysis with 1 × 10^7^ colony forming units (CFU) in 200 μl PBS. Briefly, intraperitoneal injection of 4% chloral hydrate (10 μl/g) was used to deeply anesthetize mice. The mice were kept to prone position. Colon was inserted with paraffin oil applied plastic hose (2 mm internal diameter) through anus. When the top of hose was inserted 4 cm from anus, the mice were kept to handstand. The hose was pulled out after bacterium was injected. Anus was pressed with cotton swab within 1 min, and the mice were put back to cage. Mice were separated into 6 groups: (Water) control group, water drinking and no bacterial inoculation; (DSS) DSS drinking and no bacterial inoculation; (*E. rectale* + water) water drinking and *E. rectale* inoculation; (*F. nucleatum* + water) water drinking and *F. nucleatum* inoculation; (*E. rectale* + DSS) DSS drinking and *E. rectale* inoculation; (*F. nucleatum* + DSS) DSS drinking and *F. nucleatum* inoculation. Each group consisted of five mice. The mice were monitored and weighed daily until they had lost > 20% of their initial body weight, And upon being sacrificed, the colon and spleen were both harvested for determination of colon length, histological examination and spleen weight. Stool was collected and measured daily for the presence of occult and gross blood. Occult blood was measured by a qualitative fecal occult blood detection kit.

### Histological examination

Harvested colons were cleaned in physiological saline solution to remove fecal residue. After 24 h of fixation in 10% buffered formalin, the colons were embedded in paraffin and sectioned. Then the tissues were stained with hematoxylin and eosin (H&E). Images of histology slides were taken by an Olympus IX51 microscope. Histology of colon was scored on a scale of 0–5 where 0 = normal and 5 = severe inflammation and complete loss of surface and crypt epithelium.

### Isolation of bacterial endotoxins

Endotoxic LPSs were isolated from bacteria using the hot phenol-water method as previously described [36]. In brief, bacterial pellets were digested with lysozyme. Then, cell lysate was incubated with DNase and RNase to digest nucleic acids. 90% phenol extraction procedure was used. The water layer was dialyzed against deionized water and digested with proteinase K. Then the solution was dialyzed again. The purity of the phenol extract was tested by the detection of nucleic acid (260 nm), coomassie brilliant blue stained protein (595 nm). Isolated LPSs were also detected by SDS-PAGE. Finally, LPSs were lyophilized using Freeze Dry System (FD-1A-80, BILON Ltd., Shanghai, China). The biological effectiveness of isolated LPSs was tested in Limulus Amebocyte Lysate assay according to the manufacturer’s instructions.

### LPS treatment to human normal colon epithelial cells

The human normal colon epithelial cell lines NCM460 and HCoEpiC were both purchased from Tong Pai Technology, Inc. (Shanghai, China). These cells were cultured in RPMI 1640 medium supplemented with 10% FBS, 100 μg/mL streptomycin, and 100 U/mL penicillin. LPS was dissolved in RPMI 1640 medium and vortexed for 10 min by ultrasonic bath (Shenglan Ltd., Jiangsu, China) before use. A concentration series of LPS dilutions was used to treat cells for 2.5 h.

### Western blotting

For detection of NFκΒ p65 expression in nuclei, nuclear proteins were isolated from harvested cells using a nuclear and cytoplasmic protein extraction kit and following the manufacturer’s protocol. Western blotting was carried out as previously described [37]. Briefly, protein samples were suspended in SDS loading buffer and boiling for 5 min. Then 10 μg protein was separated by SDS-PAGE and transferred to immobilon membranes by semi-dry blotting method. The membranes were probed with antibodies using standard techniques. Finally, the protein bands were visualized using ECL Plus and exposed film. Each assay was carried out in triplicate.

### Immunocytochemistry analysis

Immunofluorescence staining was used to analyze the location of NFκΒ p65 expression. The procedure was carried out as previously described [37]. In brief, cells were washed with PBS for three times, fixed in 10% formalin at room temperature for 20 min, treated with 0.5% Triton X-100 for 5 min at 4 °C and blocked with 5% normal goat serum overnight at 4 °C. Then the slides were incubated with primary antibody for 1 h at 37 °C. After washing with PBS, the slides were incubated with FITC-conjugated secondary antibody and DAPI simultaneously for 1 h, at 37 °C. Subsequently, the slides were washed with PBS and then sealed. The slides were photographed immediately using a fluorescence microscope (Olympus, Tokyo, Japan).

### Statistical analysis

All data represent mean ± standard deviation. Statistical analysis was carried out by Student’s t test using SPSS software. P value < 0.05 was considered statistically significant.

## Results

### Decreased alpha-diversity and altered overall microbial composition in location of severe lesion intestinal epithelium

**Fig. 1:**
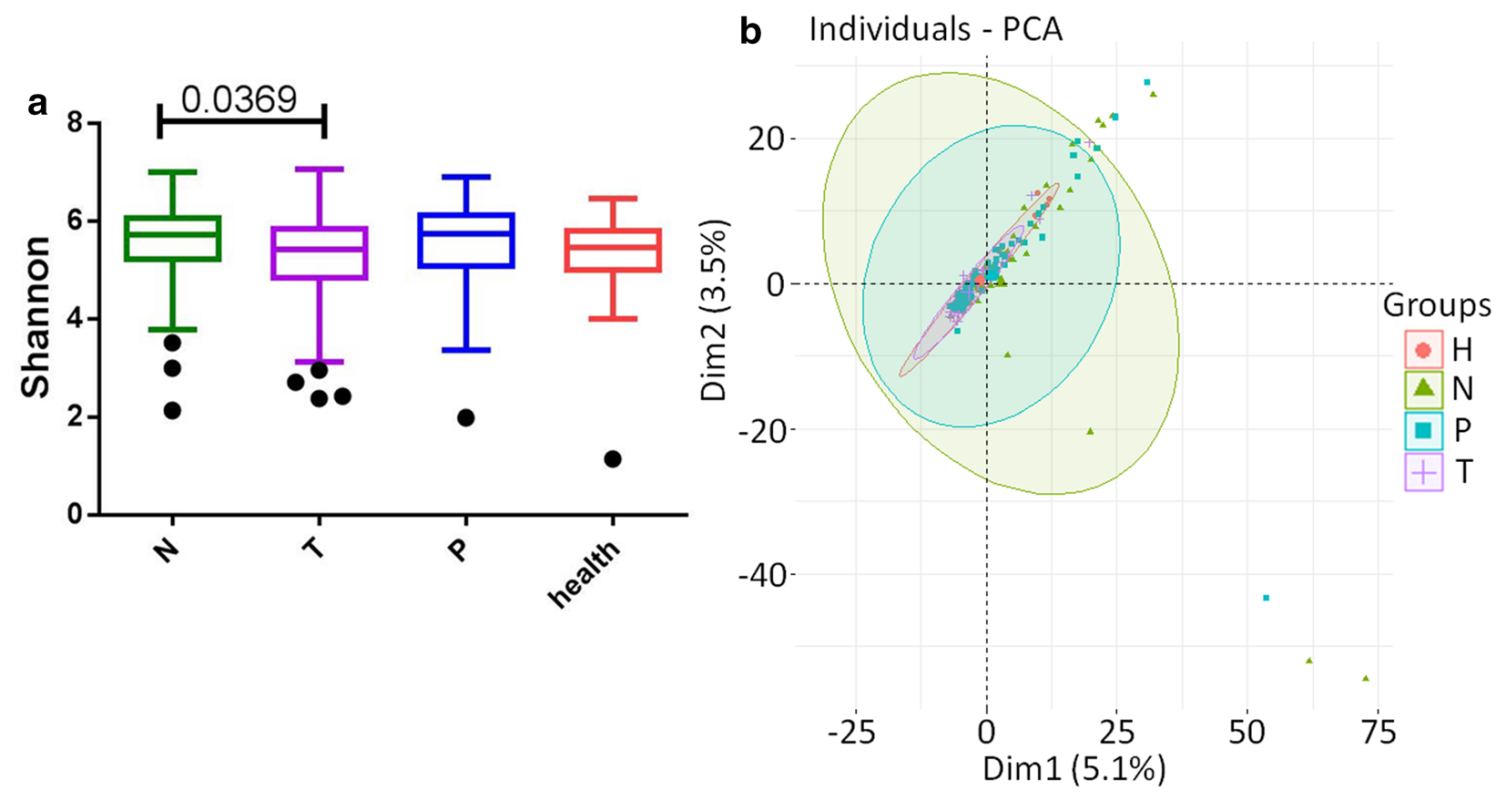
Gut microbiome biodiversity of CRC patients and healthy controls. **a** Shannon diversity index was significantly reduced in on-tumor sites compared to off-tumor sites. P value was tested using one-tailed student’s t test. **b** PCA analysis showed that the overall gut microbiota composition was different among on-tumor sites, adjacent-tumor sites, off-tumor sites, and healthy controls

To reveal microbiome profile differences among gut mucosal phenotypes, we performed 16S ribosomal RNA gene sequencing on biopsy samples collected from on-tumor sites (n = 75), tumor-adjacent sites (n = 75), and off-tumor sites (n = 75) of CRC patients, and healthy people (n = 26) at Tianjin Union Medical Center (Table 1). In the discovery cohort, severity of pathological changes in intestinal epithelium was strongly associated with a decrease of intraindividual diversity of on-tumor sties compared to off-tumor sites, as measured by Shannon diversity index (p < 0.05) (Fig. 1a). Alpha-diversity in microbiome profiles may be affected by individual difference. It has been reported that Shannon diversity index is similar in fecal samples of normal, adenoma and cancer group [12], but it is also found that bacterial diversity of gut mucosal samples is lower in CRC group than normal group [13]. To assess the overall diversity of microbiome profiles, we performed principal component analysis (PCA) based on genera abundances in different mucosal sites from CRC patients and healthy people. Figure 1b showed the microbial composition differences in different mucosal sites of patients and healthy controls.

**Table 1:** Summary information of CRC patients and healthy people

Position	CRC patients (75)		Healthy volunteers (26)	
	Rectum	Colon	Rectum	Colon
No	43	32	20	6
Male/female	29/14	19/13	14/6	3/3
Age (mean, range)	63.9 (29–81)	62.8 (35–82)	50.2 (21–71)	56.5 (48–68)
Dukes	A (3), B (23), C (17)	A (4), B (14), C (14)		

A total of 101 subjects were included in this study: 75 individuals in the CRC group and 14 individuals in healthy group. The mean age of the subjects was 63.4 ± 11 years in CRC and 51.7 ± 11.9 years in healthy group

### Bacterial differential abundances in different mucosal sites of patients and healthy controls

**Fig. 2:**
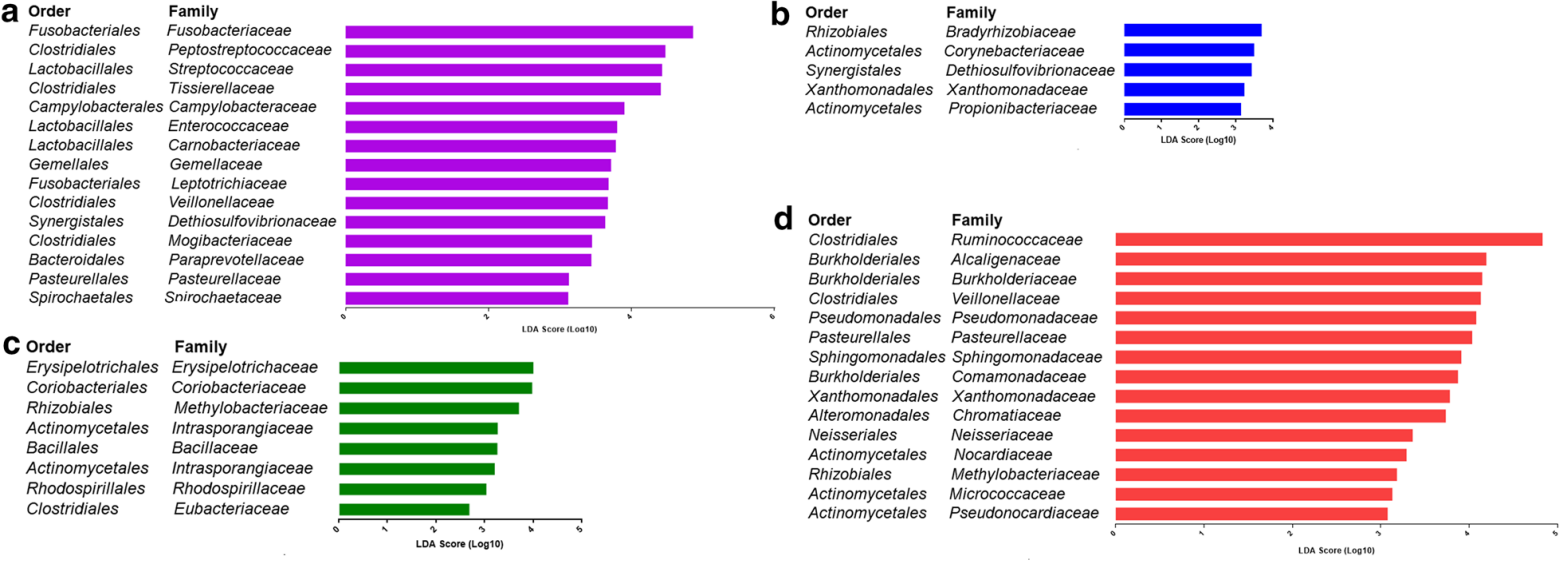
LDA analysis showed the site-specific bacterial markers in on-tumor sites (**a**), adjacent-tumor sites (**b**), and off-tumor sites (**c**) of CRC patients and healthy controls (**d**)

Using the reference Greengenes database, clustered operational taxonomic units (OTU) were assigned for bacterial phylotypes. Consistent with previous findings [13], we observed the same major bacterial phyla across the different mucosal sites of patients and healthy controls, including Firmicutes, Bacteroides, and Proteobacteria. To identify differentially abundant taxa in mucosal phenotypes as site-specific bacterial taxon markers, we performed linear discriminant analysis (LDA) on the microbial compositions of the different mucosal sites from CRC patients and healthy people (LDA score > 2.5, p value < 0.05). No significant changes were observed at phylum level among different mucosal sites from CRC patients and healthy people. In total, 15, 5, 8, and 15 families were identified to be associated with on-tumor site, adjacent-tumor site, off-tumor site, and healthy control, respectively (Fig. 2). At family level, *Fusobacteriaceae* showed the strongest correlation with on-tumor site (LDA score = 4.86, p value = 3.28E-07) (Fig. 2a), consistent with previous findings [5]. *Bradyrhizobium* (LDA score = 3.71, p value = 1.32E-11) and *Corynebacteriaceae* (LDA score = 3.49, p value = 1E-04) showed the strongest correlation with tumor-adjacent sites of CRC patients, distinct different from the taxon markers for on-tumor sites (Fig. 2b). *Erysipelotrichaceae* (LDA score = 4.02, p value = 0.03) and *Ruminococcaceae* (LDA score = 4.84, p value = 0.03) showed the strongest correlation with off-tumor sites and healthy controls, respectively (Fig. 2c and d). Our data suggest that microbial compositions in different mucosal sites and healthy controls show distinct microbial profiles. In addition, bacteria associated with off-tumor site, including the family *Eubacteriaceae*, may serve as driver bacteria that initiate CRC.

### *E. rectale* is a potential driver bacterium for CRC initiation

**Fig. 3:**
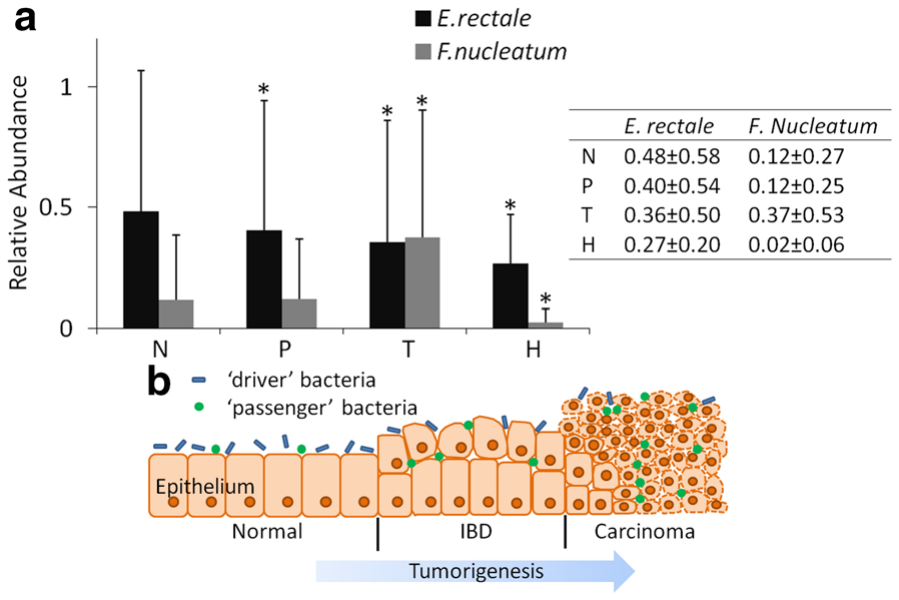
Abundance of bacteria in patient samples and diagrammatic sketch of colon epithelium. **a** Abundance of *E. rectale* and *F. nucleatum* at different sites on the colon in samples of 75 patients and 26 health people is shown as mean ± STD. “N” represents normal epithelia position of patients, “P” represents para-tumor epithelia position of patients, “T” represents tumor surface of patients, “H” represents healthy people. Other groups were compared with group “N” separately using Student’s t test. *P < 0.05 represents a significant difference from the value in the “N” group. **b** During tumorigenesis, abundance of ‘driver’ bacteria, which contributed to tumor initiation, decreased gradually owing to the high competition from the ‘passenger’ bacteria in IBD or tumor niche

Among the eight bacterial families associated with off-tumor site of CRC patients, the family *Eubacteriaceae* contains a butyrate-producing genus *Eubacterium*. Here we chose *E. rectale* as an example to examine the role of *Eubacterium* in CRC initiation. As shown in Fig. 3a, abundances of *E. rectale* and *F. nucleatum* were calculated by QIIME. As a typical bacterium associated with CRC, according to previous reports [8], *F. nucleatum* exhibits high abundance in intestinal tract of CRC patients and is practically nonexistent in samples from healthy people. Furthermore, tumor location has the highest abundance of *F. nucleatum* among all three locations of patients’ intestine, suggesting that *F. nucleatum* is suitable to grow in the tumor microenvironment. It has been reported that *E. rectale* is commonly distributed in healthy intestinal tract, and is less abundant in CRC patients [14]. Our results showed that there was lower abundance of *E. rectale* in on-tumor site of CRC patients than off-tumor site, as well as that *E. rectale* exhibits higher abundance in on-tumor site of CRC patients than healthy people samples. Local intestinal microenvironment of off-tumor sites of CRC patients is expected to be more like that of healthy people samples than that of on-tumor sites of CRC patients. Higher abundance of *E. rectale* in off-tumor site compared with healthy people may be due to other reasons, such as eating habits or individual difference. This difference in *E. rectale* abundance may also be due to its presence in these people’s intestinal tract before CRC onset, which is also one reason for CRC initiation. Therefore, we speculate that *E. rectale* may be a ‘driver’ bacterium, and next investigated the role *E. rectale* in CRC initiation (Fig. 3b).

### *E. rectale* promotes colitis in DSS mouse model of colitis-associated cancer

**Fig. 4:**
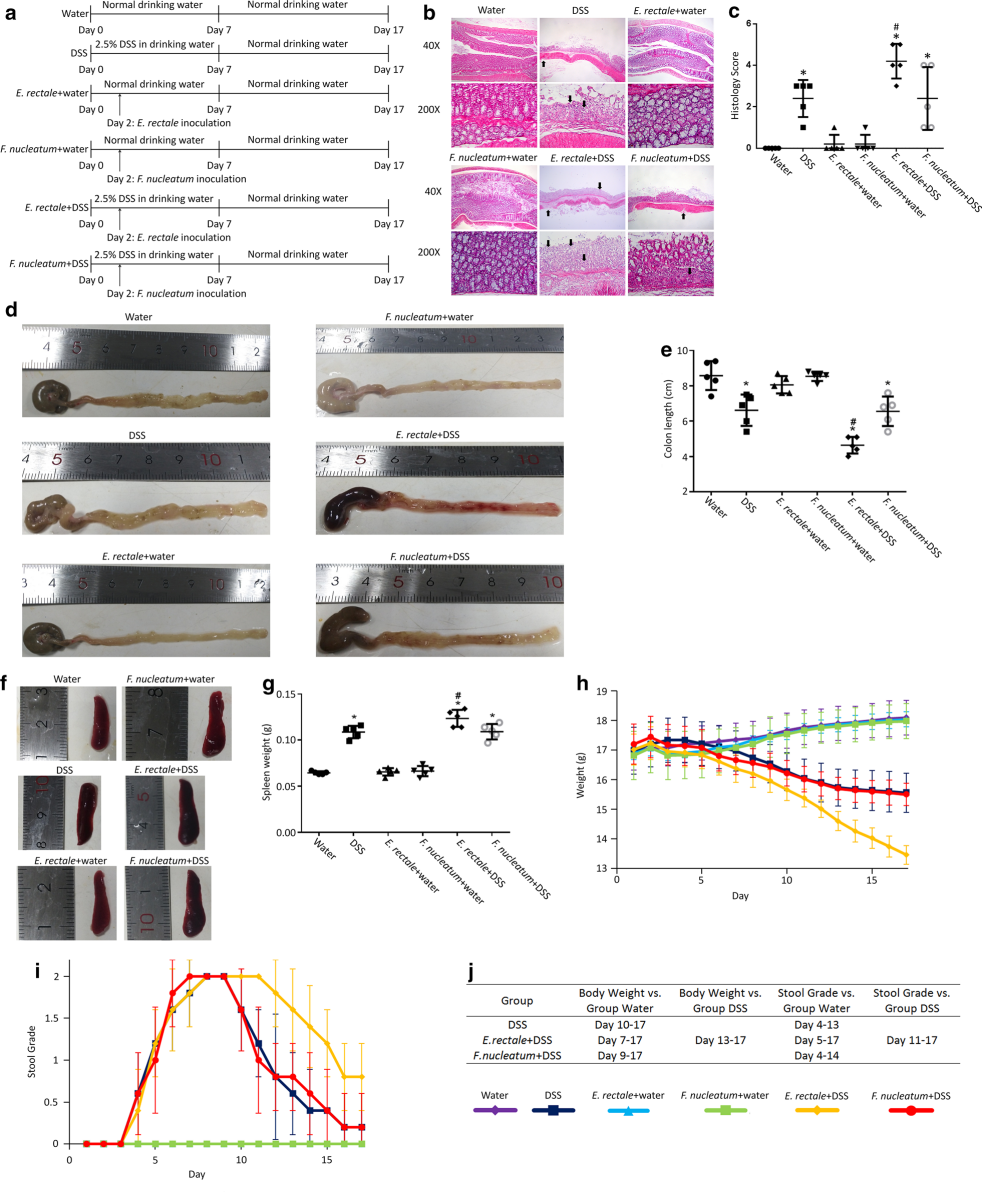
DSS induced colitis model of Balb/c mice. **a** Experimental design of seven-week-old Balb/c mouse model to evaluate the DSS exposure and bacterial inoculation. Six groups with different treatment were used, with each group having five mice. Body weight was measured daily, and organ was harvested immediately after euthanasia. **b** Representative images of H&E stained colon sections showed effect of DSS and bacterial inoculation on histopathological changes. Colon tissue from groups ‘Water’, ‘*E. rectale* + water’ and ‘*F. nucleatum* + water’ did not show any specific pathologic changes. Colon tissue from groups ‘DSS’ and ‘*F. nucleatum* + DSS’ showed rupture of the epithelium, segmental regions of marked acute inflammatory cell infiltration, ulceration, different size and shape of intestinal crypts, local destruction of crypts, which were indicated by arrows. Group ‘*E. rectale* + DSS’ had multiple crypts destruction and increased severity of the same histopathological changes seen in groups ‘DSS’ and ‘*F. nucleatum* + DSS’. **c** Graph indicated the histology scores of six groups. Data were presented as mean ± STD. *P*-values were determined using the *t*-test. Other groups were compared with group ‘Water’. Groups ‘*E. rectale* + DSS’ and ‘*F. nucleatum* + DSS’ were compare with group ‘DSS’. *P < 0.05 represents a significant difference from group ‘Water’, #*P* < 0.05 represents a significant difference from group ‘DSS’. **d**–**g** Effect of DSS and bacterial inoculation on colon length and spleen weight. Other groups were compared with group ‘Water’. Groups ‘*E. rectale* + DSS’ and ‘*F. nucleatum* + DSS’ were compare with group ‘DSS’. *P < 0.05 represents a significant difference from group ‘Water’, #*P* < 0.05 represents a significant difference from group ‘DSS’. **d** Representative gross images and measurements of mouse colons with cecums still attached. Arrows indicate the colon-cecum junction as a landmark for colon length measurement. **e** Diagram of colon length. Column indicated the mean ± STD (n = 5 mice per group). **f** Gross picture of spleen. **g** Spleen weight measured at the time of harvested. Column indicated the mean ± STD (n = 5 mice per group). **h**–**j** Body weight and stool grading of mice. Black arrow indicates the day of bacterial inoculation. Blue arrow indicates the day that mice began to drink water without DSS. Points show the mean from five mice or five stool samples, and standard deviations are shown as error bars. **h** Mice body weight alterations during the 17-day experiments. **i** Daily stool grading during the 17-day experiments. Stool grade 0 represents no occult or gross blood, stool grade 1 represents occult blood, stool grade 2 represents gross blood. **j** Other groups were compared with group ‘Water’ or group ‘DSS’. Days represent significant difference from group ‘Water’ or group ‘DSS’ (*P* < 0.05). There are no significant difference among group ‘Water’, ‘*E. rectale* + water’ and ‘*F. nucleatum* + water’

DSS mouse model of colitis was used to examine whether *E. rectale* promotes colitis which contributes to initiation of CRC. Treatment of mice in each group was shown in Fig. 4a. Histopathological analysis of H&E stained colon sections (Fig. 4b and c) revealed that, compared to the normal colonic tissue of water drinking group, DSS exposure led to segmental regions of intestinal mucosa rupture, marked acute inflammatory cell infiltration, crypt abscesses, and early architectural distortion. *E. rectale* but not *F. nucleatum* inoculation increased DSS induced colitis. Neither *E. rectale* nor *F. nucleatum* inoculation can induce obvious colitis without DSS exposure. Group ‘*E. rectale* + DSS’ showed the highest histology score, suggesting that *E. rectale* increased DSS induced colitis. Group ‘DSS’ and ‘*F. nucleatum* + DSS’ showed similar histology score, -suggesting that *F. nucleatum* did not increase DSS-induced colitis.

Figure 4d–g showed that colon length from the distal end to the colon-cecum junction and spleen weight changes. *E. rectale* or *F. nucleatum* inoculation in water drinking mice did not induce a decrease in colon length or increase spleen weight. DSS exposure caused significant decrement in colon length or increment in spleen weight, and additional *E. rectale* inoculation further increased this effect. By contrast, *F. nucleatum* inoculation did not increase the effect of DSS on colon length shorten or spleen weight increment.

As shown in Fig. 4h and j, Body weight in inoculated groups showed no significant difference with Water group. Body weight in DSS group showed significant difference starting at day 10, additional *F. nucleatum* inoculation shift this time point to day 9, and additional *E. rectale* inoculation shift this time point to day 7. Body weight in group ‘*E. rectale* + DSS’ continued to decrease until the end of experiment, but body weight in groups ‘DSS’ and group ‘*F. nucleatum* + DSS’ declined slowly from day 13 to the end of experiment, the reason of which may be due to *E. rectale* but not *F. nucleatum* inoculation enhance colitis induced by DSS. In addition, all mice survived until the end of experiment.

As shown in Fig. 4i and j, there are no occult or gross blood in stool in Water group and inoculated groups. DSS drinking groups had occult blood in stool at day 4 and gross blood in stool at day 5. These groups began to exhibit gross blood in all stool at day 8. DSS group and *F. nucleatum* + DSS group both showed a decrease in gross blood and an increase in adult blood in stool from day 10. Occurrence of occult blood in stool of these two groups also continued to decrease from day 12 to the end of experiment. Compared to these two groups, *E. rectale* + DSS group has more time of gross blood occurrence, and incidence rate of occult blood decrease slower.

### *E. rectale* LPS promotes NF-κΒ p65 expression in nuclei of intestinal epithelial cells

NF-κΒ is a critical transcription regulator that regulates the expression of many genes of inflammatory functions and the immune system. NF-κΒ activation facilitates both tumor development and metastatic progression cancer [15, 16]. NF-κΒ has two subunits, p50 and p65, to form a homodimer or heterodimer. The dimer maintains normal physiological function in vivo. In normal conditions, NF-κΒ is sequestered by members of the IκB family to form an inactivated compound in the cytoplasm. Under external stimuli, such as enteroinvasive bacterial infection or cytokine stimulation to intestinal epithelial cells [17, 18], NF-κΒ is activated. During this process, IκB kinase (IKK) is activated and caused IκB degradation, which induced NF-κΒ dissociation from IκΒ and transferred to the nucleus. Therefore, we measured expression levels of IKKα and IκBα in cytoplasm, and p65 in nucleus to determine NF-κΒ activation.

**Fig. 5:**
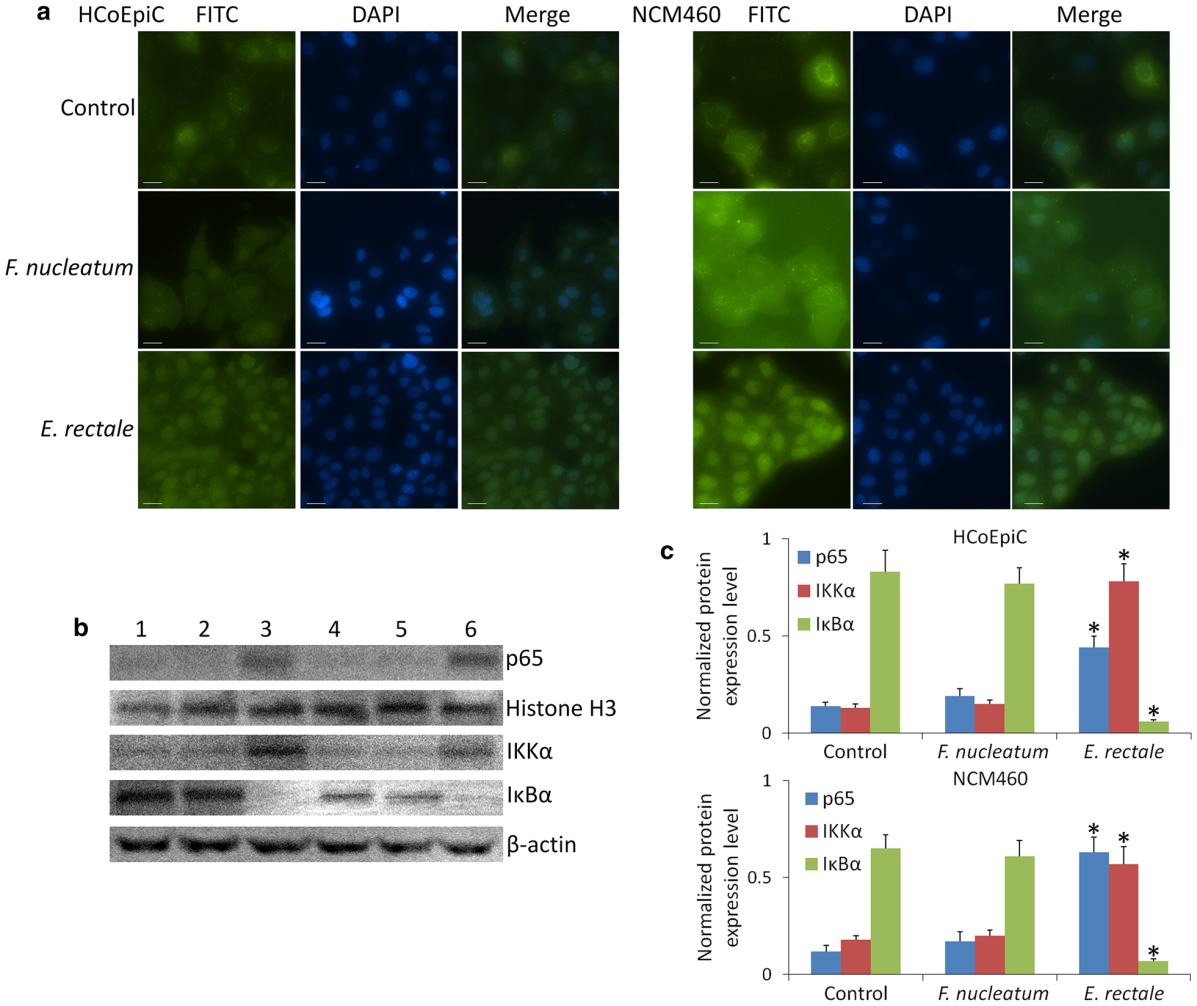
NF-κΒ p65 expression in HCoEpiC and NCM460 cells. **a** Immunocytochemistry results. There is obviously expression in nuclei of HCoEpiC and NCM460 cells treated by 20 ng/ml of LPS extracted from *E. rectale* for 2.5 h but not *F. nucleatum*. Untreated cells were used as control group and exhibited p65 expression in plasma. **b** Western blot results. 1 and 4: No treated cells; 2 and 5: 20 ng/ml of *F. nucleatum* LPS treatment; 3 and 6: 20 ng/ml of *E. rectale* LPS treatment. Two cell lines were measured including: 1–3: HCoEpiC; 2–6: NCM460. **c** The densitometric analysis bar diagram of the results. p65 expression level was normalized by Histone H3 expression level. IKKα and IκBα expression level were normalized by β-actin expression level. Columns represent the mean from three independent experiments and bars represent standard deviations. *P < 0.05, significantly different from control group

Two human normal colon epithelial cell lines, HCoEpiC and NCM460, were used to investigate whether *E. rectale* LPS induces inflammatory in vitro. Our results (Fig. 5) showed that NF-κΒ is activated in both HCoEpiC and NCM460 when they were treated by 20 ng/ml of *E. rectale* LPS for 2.5 h. NF-κΒ was significantly expressed in nuclei of HCoEpiC and NCM460 cells after treating with *E. rectale* LPS. By contrast, *F. nucleatum* LPS does not show this effect at the same concentration. Purity measurement of LPS is shown in Additional file 1: Figure S4.

## Discussion

According to the ‘driver-passenger’ model proposed by Tjalsma et al. [9], CRC ‘driver’ bacteria may not preferentially inhabit on-tumor sites, because many ‘passenger’ bacteria have high adaptability on-tumor sites and colonize most of the on-tumor sites niches. Conversely, the off-tumor site niches may be maintained similar to that on healthy intestinal tract. Thus, ‘driver’ bacteria may still be present in considerable abundance in off-tumor sites during CRC tumorigenesis [19]. We found that *E. rectale* distribution in our samples fit the characteristic of ‘driver’ bacteria, leading to our further investigation on the contribution of *E. rectale* to colitis in vitro and in vivo.

*F. nucleatum* is anaerobic Gram-negative bacterium, which is an adherent and invasive species. It is very suitable to survive in the broken colon wall and has high abundance in IBD [20] or CRC [6] samples. Because *F. nucleatum* is a potential ‘passenger’ bacterium [9], it is used as control bacterium in this study. Many reports found that *F. nucleatum* has higher abundance in CRC patients biopsy or fecal samples measured by 16S ribosomal RNA profiling [21, 22]. Our results also exhibited that abundance of *F. nucleatum* increased in CRC patient samples, especially in on-tumor sites.

*E. rectale* is an anaerobic Gram-positive bacterium and is one of the most abundant bacterial species in human fecal samples. Some reports showed that *E. rectale* is a major species for butyrate formation, which is the preferred energy source for colonocytes and benefits for colon health [23], but butyrate also promotes CRC by stimulating the transformation of colonic epithelial cells and inducing aberrant proliferation [2]. *E. rectale* is reported to be significantly less present [24] in ulcerative colitis (UC) patients, which implies that *E. rectale* may initiate colitis that leads to CRC initiation if *E. rectale* is a ‘driver’ bacterium. According to this report, *E. rectale* seems to function as probiotic, but some recent studies that focused on distribution of intestinal microflora in CRC samples implied that *E. rectale* may be a potential ‘driver’ bacterium [7, 9]. Butyrate-producing bacteria, including *Eubacterium*, were found to be less abundant in CRC fecal microbiota measured by 16S ribosomal RNA profiling [12]. To our best knowledge, the studies about *E. rectale* abundance in CRC samples measured by 16S ribosomal RNA profiling are lacking. There are only a few reports [22] on which other species in the genus *Eubacterium* were measured by 16S ribosomal RNA profiling in CRC samples, such as *E. hallii*, *E. oxidoreducens* and *E. ruminantium*, which exhibited different trend of abundance changes in carcinogenesis process, suggested that different species in the genus *Eubacterium* have different adaptabilities to the on-tumor site niche. Our 16S ribosomal RNA profiling also showed that *E. rectale* has considerable density in healthy samples and higher abundance in off-tumor sites, but abundance of *E. rectale* declined in on-tumor sites significantly. Thus, we further investigated whether *E. rectale* contributes to CRC initiation by promoting inflammation in vitro and in vivo.

Cancer is a disease mainly caused by genetic mutations, but inflammation can temporarily bypass the mutation requirement for tumor initiation. Besides mutations, IBD is an important risk factor for the development of CRC. Two of the most common forms of IBD are Crohn’s disease (CD) and ulcerative colitis (UC). For example, Crohn's disease (CD) increases cumulative risk of colitis associated cancer (CAC) by up to 8% [25], and Ulcerative colitis (UC) up to 18–20% risk of CAC [26]. Wnt/β-catenin, which is a critical pathway to regulate normal and malignant cell proliferation, is activated by mutation in over 90% of sporadic CRC [27], which including adenomatous polyposis coli (APC). But several inflammatory pathways, including NF-κΒ, can induce Wnt/β-catenin activation without any mutations in APC [28].

Some of the symbiotic or commensal intestinal bacteria in human beings and mice have been found as conditionally pathogenic, which induces various forms of IBD. For example, *Helicobacter spp.* infection in Il10^−/−^ mice usually induces IBD and consequently CAC development [29]. Under normal conditions, intestinal bacteria are separated from immune system by complete protective epithelial barrier. When intestinal epithelial barrier is breakdown by some factors, for example DSS, commensal flora translocates to interact with immune system, which causes IBD development. Many potential ‘driver’ bacteria induce IBD, such as *Bacteroides fragilis* [10, 11] and *Clostridium difficile* [30]. Several reports show that abundance of *Fusobacterium nucleatum* [20] increases and *E. rectale* declines [14] in UC patients. Although *F. nucleatum* is also enriched in CRC samples and appears to be the most common passenger bacterium [5], this does not constitute sufficient evidence to confirm that it plays an active part in IBD or CRC progression [7]. And the relation between *E. rectale* and IBD promotion is also still unknown.

## Conclusion

In our study, we investigated the relation between *E. rectale* and IBD promotion by utilizing the well-established dextran sulfate sodium (DSS) model of colitis in Balb/c mouse. This model was widely used to mimic characteristics of IBD, induce acute colitis and sustain chronic levels of inflammation [31]. This model has been further verified by applications of several therapeutic agents used to treat human IBD [32]. We found that *E. rectale* but not *F. nucleatum* prolonged and/or worsened the severity of DSS-induced colitis, and *E. rectale* also activated inflammatory factor, NF-κΒ, in normal colon epithelial cells. Combined with *E. rectale* distribution in our samples, our findings suggest that *E. rectale* is a ‘driver’ bacterium and contribute to CRC initiation by promoting colitis.

## Supplementary Information

**Additional file 1.** Additional figures.

## Data Availability

The 16S rRNA sequencing reads have been submitted to the NCBI SRA database under accession number PRJNA606879. All the data are provided in this manuscript and supplementary materials.

## Abbreviations

CRC: Colorectal cancer
IBD: Inflammatory bowel disease
PAMP: Pathogen-associated molecular patterns
LPS: Lipopolysaccharide
ETBF: B. fragilis
BFT: Metalloprotease toxin
LefSe: Linear discriminant analysis Effect Size
T: Tumor position
P: Para-tumor epithelia position
N: Normal epithelia position
H: Healthy people
DSS: Dextran sodium sulfate
CFU: Colony forming units
H&E: Hematoxylin and eosin
LDA: Linear discriminant analysis
IKK: IκB kinase
