# Supplementary material for: Eubacterium rectale contributes to colorectal cancer initiation via promoting colitis
Source: Gut Pathog. 2021 Jan 12;13:2. doi: 10.1186/s13099-020-00396-z (PMC7805161; doi:10.1186/s13099-020-00396-z)
Supplement: Supplementary file 1 — Additional file 1. Additional figures. [file 13099_2020_396_MOESM1_ESM.pdf]

Supplementary materials

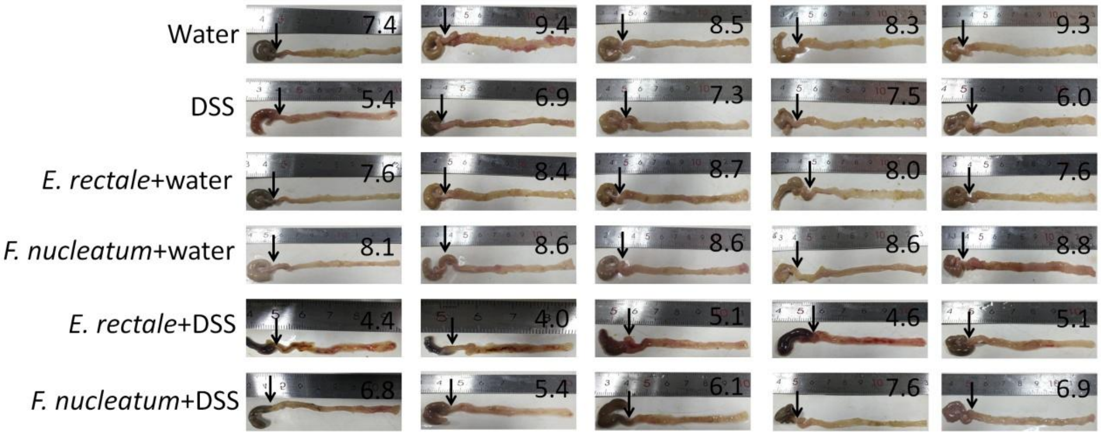

Figure S1. Gross images and measurements of mouse colons with cecums still attached. Arrows indicate the colon-cecum junction as a landmark for colon length measurement. The left column of numbers represents group numbers which were defined in section 2.5. Numbers in each small picture represents colon length (cm).

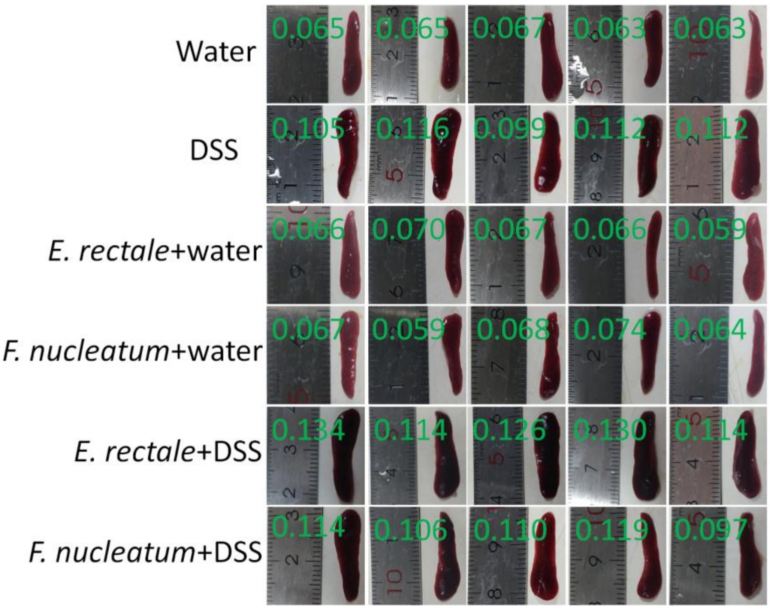

Figure S2. All gross images and measurements of mouse spleen. The left column of numbers represents group numbers which were defined in section 2.5. Numbers in each small picture represents spleen weights (g).

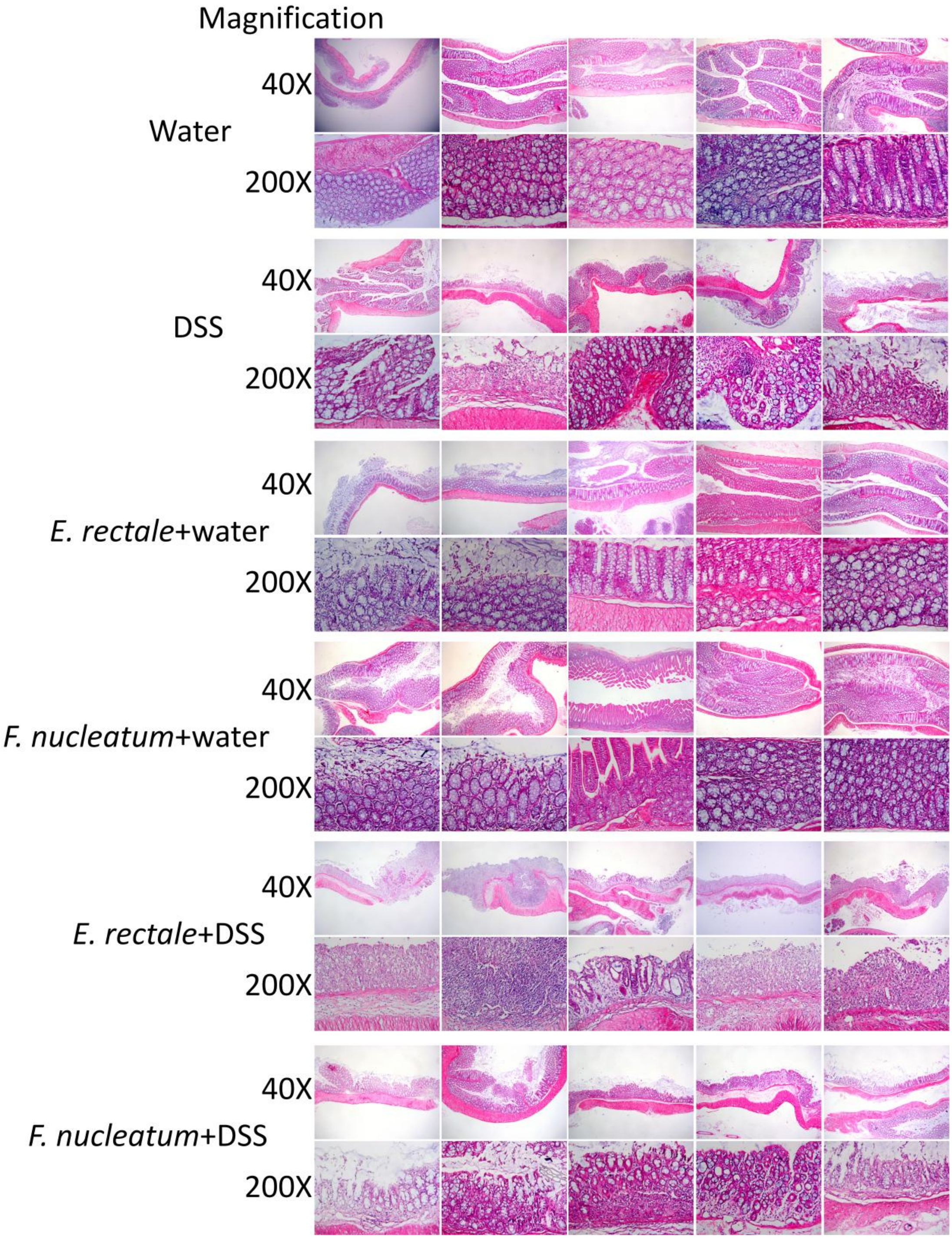

Figure S3. All H&E staining results.

A

LPS	nucleic acid (%)	protein (%)
E. rectale	24.68	1.47
F. nucleatum	28.11	1.84

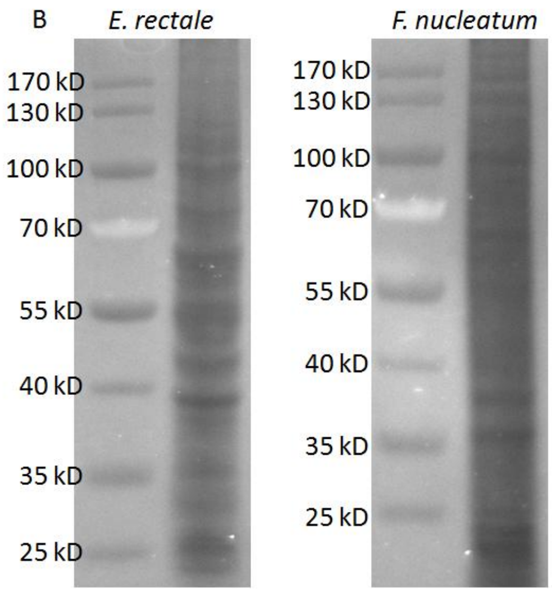

Figure S4. Measurement of phenol extraction (LPS). (A) The content of nucleic acid and protein in LPS of phenol extraction. (B) 30 μg LPS was separated by SDS-PAGE.

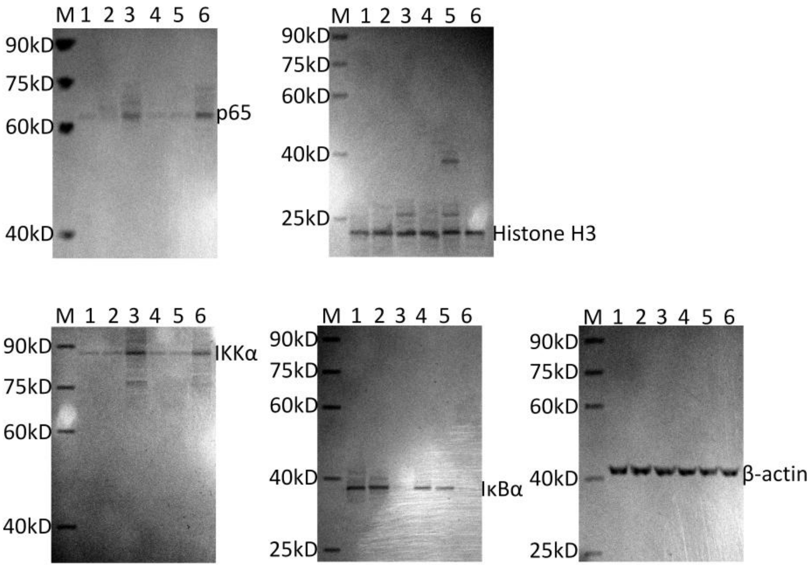

Figure S5. Full length blots of Figure 5.
